# Long-term natural history of ellipsoid zone width in USH2A-retinopathy

**DOI:** 10.1136/bjo-2024-325323

**Published:** 2024-08-05

**Authors:** Michael Heyang, Joshua L Warren, Paulina Ocieczek, Jacque L Duncan, Mariya Moosajee, Lucian V Del Priore, Liangbo Linus Shen

**Affiliations:** 1 Department of Ophthalmology and Visual Science, Yale School of Medicine, New Haven, Connecticut, USA; 2 Department of Biostatistics, Yale University School of Public Health, New Haven, Connecticut, USA; 3 Moorfields Eye Hospital NHS Foundation Trust, London, UK; 4 Department of Ophthalmology, University of California San Francisco, San Francisco, California, USA; 5 Institute of Ophthalmology, University College London, London, UK

**Keywords:** Imaging, Retina, Degeneration, Epidemiology

## Abstract

**Aims:**

To investigate the long-term natural history of ellipsoid zone (EZ) width in *USH2A*-retinopathy.

**Methods:**

EZ width measurements from optical coherence tomography were retrospectively obtained from 110 eyes of 55 participants with molecularly confirmed biallelic *USH2A*-retinopathy. We used a hierarchical Bayesian method to construct and compare different mathematical models describing the long-term decline of EZ width.

**Results:**

Compared with linear and quadratic models, exponential decline best represented the long-term loss of EZ width based on the deviance information criterion score. Log-transformed EZ width declined linearly over 30 years of inferred disease duration (median: 0.063 (IQR: 0.040–0.086) log (µm)/year). Compared with the raw EZ width decline rate, the log-transformed EZ width decline rate required 48% fewer patients to achieve an identically powered 1-year trial (38 vs 73 participants). Log EZ width decline rate was uncoupled from baseline EZ width (Spearman ρ=−0.18, p=0.06) and age (ρ=−0.10, p=0.31). Eyes with Usher syndrome exhibited earlier median onset ages of macular EZ width loss (18.8 (IQR: 13.1–24.7) vs 28.1 (IQR: 18.5–35.8) years, p<0.001) but comparable log EZ width decline rates (0.060 (IQR: 0.035–0.100) vs 0.065 (IQR: 0.050–0.079) log (µm)/year; p=0.42).

**Conclusions:**

EZ width follows an exponential decline in *USH2A*-retinopathy. Compared with raw EZ width decline rate, log-transformed EZ width decline rate may be a superior endpoint for clinical trials. Syndromic eyes exhibit an earlier onset of macular EZ width loss but progress at comparable rates to non-syndromic eyes.

**WHAT IS ALREADY KNOWN ON THIS TOPIC**

**WHAT THIS STUDY ADDS**

- The width of the ellipsoid zone declines exponentially over time; log transforming the ellipsoid zone width decline rate improves power characteristics and removes dependence on some baseline confounders.

**HOW THIS STUDY MIGHT AFFECT RESEARCH, PRACTICE OR POLICY**

- The log-transformed ellipsoid zone width decline rate should be preferred over the raw rate as an anatomic endpoint for future clinical trials.

## Introduction

 Retinitis pigmentosa (RP) is a group of diseases characterised by progressive and concentric loss of retinal photoreceptors, affecting roughly 1 in 4000 individuals.^1^ Mutations in *USH2A* account for approximately 15% of autosomal recessive non-syndromic retinitis pigmentosa cases^1 2^ and are most frequently implicated in Usher syndrome type II (USH2), a syndromic form of RP characterised by concurrent congenital sensorineural hearing loss.^2 3^ *USH2A*-retinopathy currently has no treatments approved by any major pharmaceutical regulatory agencies but several potential treatments including gene therapies, antisense oligonucleotides and neuroprotective agents are under investigation.^2^ To determine the efficacy of potential therapies, it is essential to determine the underlying natural history of disease progression and to establish robust and sensitive clinical trial endpoints for ongoing and upcoming clinical trials.

The best-corrected visual acuity is the primary endpoint in most clinical trials in *USH2A*-retinopathy, but it cannot adequately capture early disease progression as central vision usually remains unaffected until later disease stages.^4^ The ellipsoid zone (EZ) width measured by optical coherence tomography (OCT) is a promising anatomical endpoint given the strong correlation with functional criteria such as visual acuity^5^ and visual fields^6^ as well as significant and measurable annual progression.^7^ However, longitudinal data on EZ width in *USH2A*-retinopathy remains sparse in the literature so the long-term decline pattern of EZ width in these patients is unclear.^8 9^ Several studies that have investigated the change of EZ width in various other types of RP have posited exponential decay of EZ width and reported the annualised percentage of loss, given that higher EZ width decline rates were typically associated with younger age or higher baseline EZ width.^710^,^13^ Other studies have reported annualised decline rate of EZ width (in µm/year), assuming a linear decline.^9 14^ To date, there has been no clear consensus for preferring one model over the other and many studies report both outcomes in patients with RP.^7 10^ Since these studies were limited by relatively short follow-up durations, it is difficult to distinguish exponential decline from linear decline in these datasets.^7^

It is challenging to directly observe a cohort of patients with retinal degenerations over decades and directly determine the multidecade progression of these diseases. We have recently developed Bayesian entry time realignment (BETR),^15^ a hierarchical Bayesian modelling framework to determine the long-term natural history of chronic diseases by analysing data from many patients, enrolled at different points in their natural history, over shorter durations. Patients are typically enrolled in clinical studies at different stages of their disease (ie, different entry times) and often exhibit different disease progression rates. BETR adjusts for these heterogeneities by estimating an inferred disease duration and progression rate for each patient, which can then realign individual patients’ datasets to reconstruct the long-term disease course. BETR also allows objective comparisons between different competing models of disease progression and predicts unique disease trajectories for each patient, allowing detailed comparisons of disease course between different subgroups. Therefore, we aimed to use BETR to investigate the long-term progression model of EZ width in USH2A-retinopathy.

## Methods

### Study population and imaging

**Figure 1:**
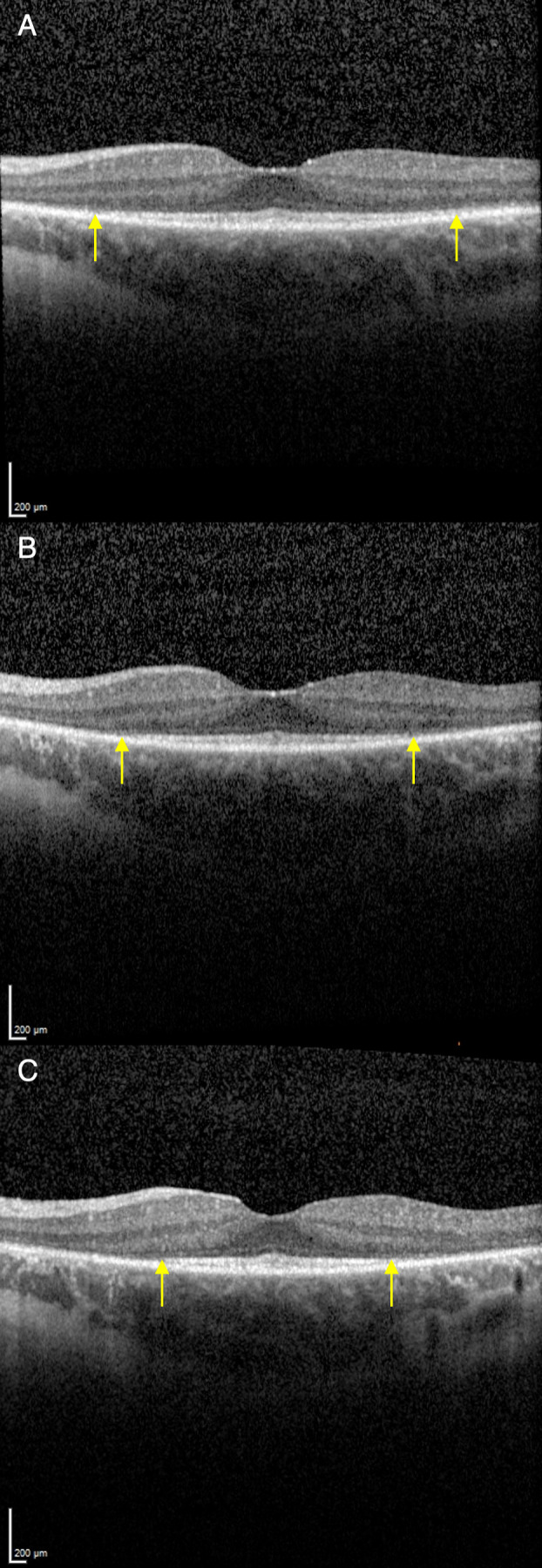
Representative set of spectral-domain optical coherence tomography (OCT) scans obtained over three longitudinal visits from a single study participant. We measured the width of the ellipsoid zone (EZ) band (marked by yellow arrows) at each visit. (A) OCT scan at baseline visit; EZ width=3900 µm. (B) OCT scan 3 years after baseline visit; EZ width=3337 µm. (C) OCT scan 4.75 years after baseline visit; EZ width=2912 µm.

Our study included 55 patients (110 eyes) with biallelic *USH2A* mutations from Moorfields Eye Hospital National Health Service Foundation Trust, London, UK, previously identified and described in a retrospective analysis by Toms *et al*.^16^ Briefly, each patient had three longitudinal clinic visits (approximately 15 months apart) with OCT imaging of both eyes, and a residual EZ width (figure 1) of at least 500 µm on baseline OCT but without extension beyond the image borders. We excluded one patient from the original cohort due to the lack of OCT imaging. We obtained 20°×20° spectral domain OCT scans (19 B-scans with 512 A-scans per B-scans or 97 B-scans with 1024 A-scans per B-scan; 9 frames per section) using the Heidelberg Spectralis (Heidelberg Engineering, Heidelberg, Germany) and assessed quality based on the absence of eye movement, blink artefact, and media opacity, and the presence of foveal centration. Measurements of the EZ width were obtained from the central OCT scan by two trained graders using the Heidelberg Eye Explorer Region Finder V.2.4.3.0.

### Data analysis

We compared three disease progression models for EZ width decline in *USH2A*-retinopathy: linear, quadratic and exponential decline (online supplemental figure 1). We first plotted EZ width as a function of time after enrolment for all eyes. However, participants had different and unknown durations of disease at enrolment (ie, different entry times) and the long-term progression pattern was unclear. To correct for differences in patients’ entry time into the study, we applied BETR to infer each eye’s baseline duration of any macular EZ width loss (in years), defined as the time interval from when the EZ width was 6000 µm (approximately the diameter of the macula, corresponding to a 20° OCT scan) to when it decreased to the observed EZ width at the baseline visit. We then added the inferred disease duration to each timepoint, essentially converting the horizontal axis from time after enrolment to the inferred duration of macular EZ width loss. We calculated the onset age of macular EZ width loss as the age at baseline minus the inferred duration of macular EZ width loss. The BETR calculations were contingent on the underlying, user-specified mathematical model for disease progression so we repeated this analysis for all three EZ width progression models. We determined the best model by calculating each model’s deviance information criterion (DIC), which is a commonly used Bayesian model comparison tool that balances model fit and complexity.^17^ A lower DIC indicates a preferable model. We performed BETR using the ‘rjags’ package in R software; specific models and settings are detailed in online supplemental methods. We previously validated BETR using both simulated and actual clinical data,^15^ and similar approaches have been employed by us and other groups to analyse the long-term disease progression in ocular,^818^,^23^ pulmonary^24^ and neurological^25 26^ diseases.

We compared the power characteristics of EZ width decline rate and log EZ width decline rate using the ‘pwr’ package in R software. We selected eyes whose initial follow-up visit occurred 9–15 months after the baseline visit and used the measurements in the baseline and follow-up visits to calculate the annualised EZ width decline rate and log EZ width decline rate. We calculated the effect size from the mean and pooled SD of the rates and calculated the required number of eyes per group for a trial to provide 80% power to detect a 30% decrease in EZ width decline rate or the mathematically equivalent log EZ width decline rate over 1 year between two independent groups (significance=0.05; enrolment ratio=1:1).

We used the Mann-Whitney U test for comparisons of continuous, non-normally distributed variables, the Student’s t-test for normally distributed variables and the paired t-test for paired data. We calculated Pearson and Spearman correlation coefficients to assess for linear and monotonic relationships. To compare the distributions of categorical variables, we used the χ^2^ test. We calculated the EZ width decline rate for each eye by performing a univariate linear regression of EZ width at all visits. We calculated log EZ width decline rates by taking the natural log of each EZ width measurement (in log µm) and performing linear regression afterwards. For brevity, we use ‘log’ to denote ‘natural log.’ Mathematically, the log EZ width decline rate is equivalent to the exponential decay constant so we converted the log EZ width decline rate to an annual exponential percentage EZ width decline rate by the following expression: 1–exp(−[decay constant]). We calculated the half-life by dividing the natural log of 2 by the decay constant. We repeated all modelling and statistical testing using the left and right eyes separately for a sensitivity analysis to account for intereye correlations. We performed all statistical analyses using R software V.4.0.3 (R Foundation for Statistical Computing, Vienna, Austria). Significance was set to 0.05 for all statistical testing.

## Results

### Patient characteristics

This study included 110 eyes from 55 participants, 34 of whom had syndromic disease (table 1). The mean±SD age at the baseline visit was 39±11 years, and the median and IQR of the follow-up duration was 2.67 (2.29–3.17) years. The median baseline EZ width among all eyes was 2202 (IQR: 1533–3019) µm, and the median baseline visual acuity was 0.18 (IQR: 0.18–0.48) logMAR, corresponding to approximately 20/30 or 6/9 vision. The allele variants for each patient are listed in online supplemental table 1.

**Table 1:** Baseline characteristics of patients stratified by syndromic status

	Total (n=55)	USH2 (n=34)	NSRP (n=21)	P value
Baseline age, years, mean (SD)	39 (11)	36 (12)	42 (10)	0.04
Sex, female (%)	25 (45)	16 (47)	9 (43)	0.98
Race, (%)*				0.66
White	25 (45)	17 (50)	8 (38)	
Black	3 (5)	2 (6)	1 (5)	
Asian	3 (5)	1 (3)	2 (10)	
Other/unknown	24 (44)	14 (41)	10 (48)	
Baseline VA, logMAR, median (IQR)†	0.18 (0.18–0.48)	0.30 (0.18–0.48)	0.18 (0–0.30)	0.02
Baseline EZ width, µm, median (IQR)†	2202 (1533–3019)	2191 (1632–2985)	2358 (1485–3195)	0.83
Follow-up duration, years, median (IQR)	2.67 (2.29–3.17)	2.71 (2.15–3.15)	2.50 (2.34–3.17)	0.72

* White includes white British, white Irish; Asian includes Asian Indian, Asian Pakistani.

† Statistics for baseline VA and EZ width were calculated using all 110 eyes.

EZellipsoid zoneNSRPnon-syndromic retinitis pigmentosaUSH2Usher syndromeVAvisual acuity

### EZ width declines exponentially over approximately 30 years of inferred disease duration

BETR showed that the exponential decay model for EZ width had the lowest DIC score among the three competing models (exponential, −1379; linear, −839; quadratic, −614), indicating that exponential decay best described the long-term decline of EZ width. This same trend was observed when the left and right eyes were modelled separately (online supplemental table 2A).

**Figure 2:**
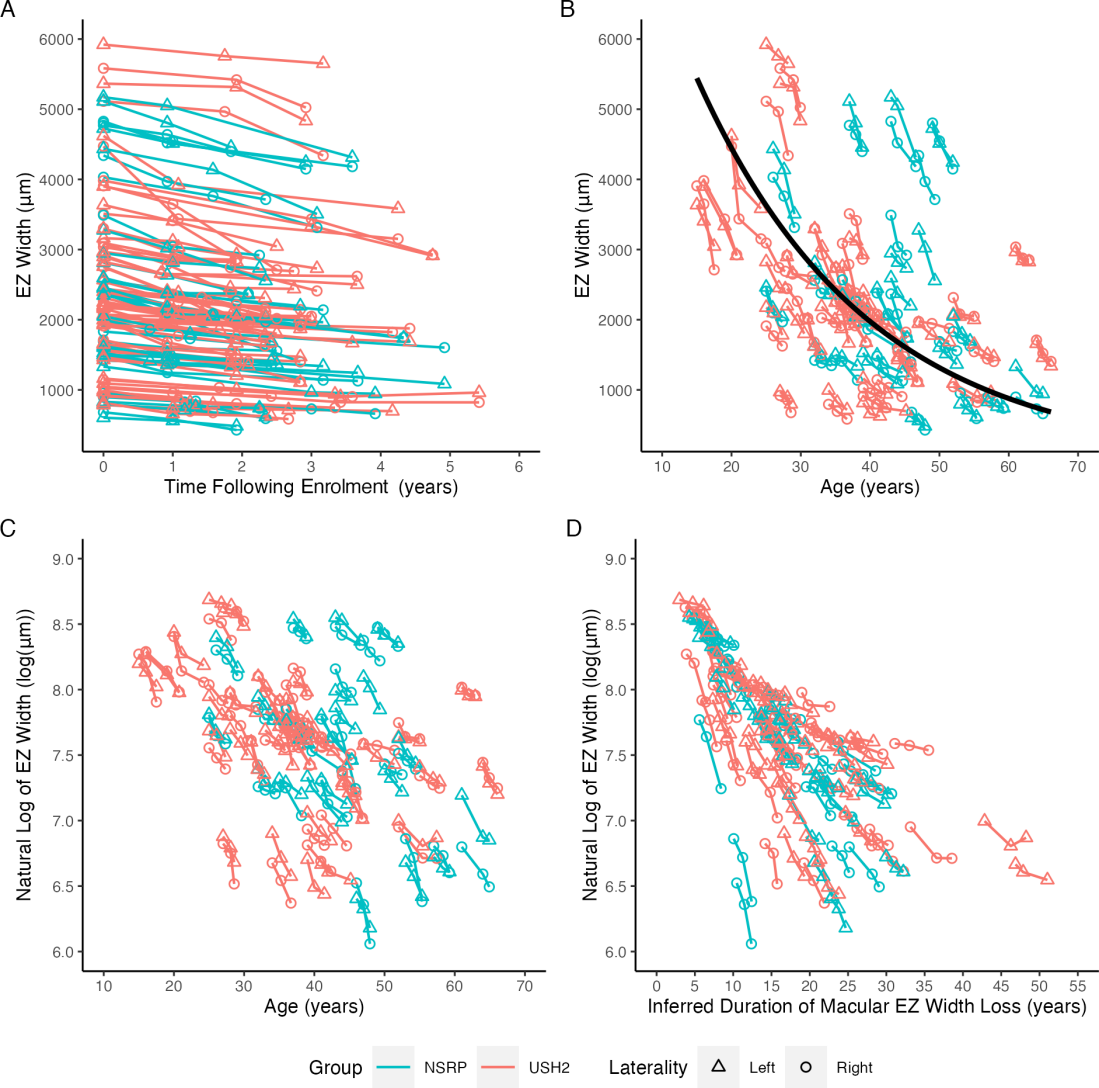
Graphical representations of ellipsoid zone (EZ) width loss over time. USH2, n=68 eyes; NSRP, n=42 eyes. (A) EZ width plotted against time following enrolment (baseline visit). Due to limited follow-up durations (<5 years), the long-term progression is not discernible. (B) Same data as (A) with EZ width plotted against age and fitted to an exponential trendline (EZ=10 000×e^−0.0406×age^). (C) Linearised version of (B) after applying natural log to EZ width. Log EZ width appears to decline linearly, although varies at the same age among different eyes, suggesting heterogeneity in the onset age of macular EZ width loss and potentially the rate of disease progression. (D) Log EZ width as a function of inferred duration of macular EZ width loss. Because the age did not sufficiently capture the disease duration, we used Bayesian entry time realignment to estimate the duration of macular EZ width loss for each eye and horizontally translated the datasets in (A) to convert the horizontal axis from time after enrolment to inferred duration of macular EZ width loss. Realignment of the data demonstrated that the log EZ width of individual eyes declined linearly at different rates over approximately 30 years. Eyes with similar log EZ width decline rates were in-line with one another, extending from a single origin at log (6000 µm). NSRP, non-syndromic retinitis pigmentosa; USH2, Usher syndrome type II.

To study the long-term natural history, we began by plotting EZ width against time after enrolment (figure 2A). Individual eyes had varying EZ widths at baseline, suggesting different stages of the disease at the time of enrolment. Figure 2B shows that EZ width appears to follow a trend of exponential decline over age, although this only loosely fits the data. We further log-transformed EZ width to linearise the data from figure 2B and plotted it as a function of age (figure 2C). Log EZ width appeared to decline linearly on a logarithmic scale, consistent with the hypothesis of exponential decline, although eyes at any given age had considerable variability in log EZ widths, indicating different onset ages of macular EZ width loss or different disease progression rates.

Because the age alone did not sufficiently capture the duration of macular EZ width loss, we performed BETR to estimate the duration of macular EZ width loss for each eye and horizontally translated raw datasets in figure 2A to convert the horizontal axis from time after enrolment to inferred duration of macular EZ width loss (figure 2D). Realignment of the data demonstrated that the log EZ width of individual eyes declined linearly at different rates over approximately 30 years. Eyes with similar log EZ width decline rates were in line with each other, extending from a single origin at log (6000 µm). The median decline rate of log EZ width was 0.063 (IQR: 0.040–0.086) log (μm)/year, equivalent to a 6.13 (IQR: 3.96–8.28) per cent annual decline rate. The estimated median age at onset of macular EZ width loss was 21.1 (IQR: 14.8–29.0) years. The median half-life, or time required for EZ width to reach exactly half of its initial value, was 11.0 (IQR: 8.0–17.2) years. The parameters estimated by BETR did not change when the left and right eyes were modelled separately (online supplemental figure 2).

### Log EZ width decline rate may be a sensitive anatomic endpoint

**Figure 3:**
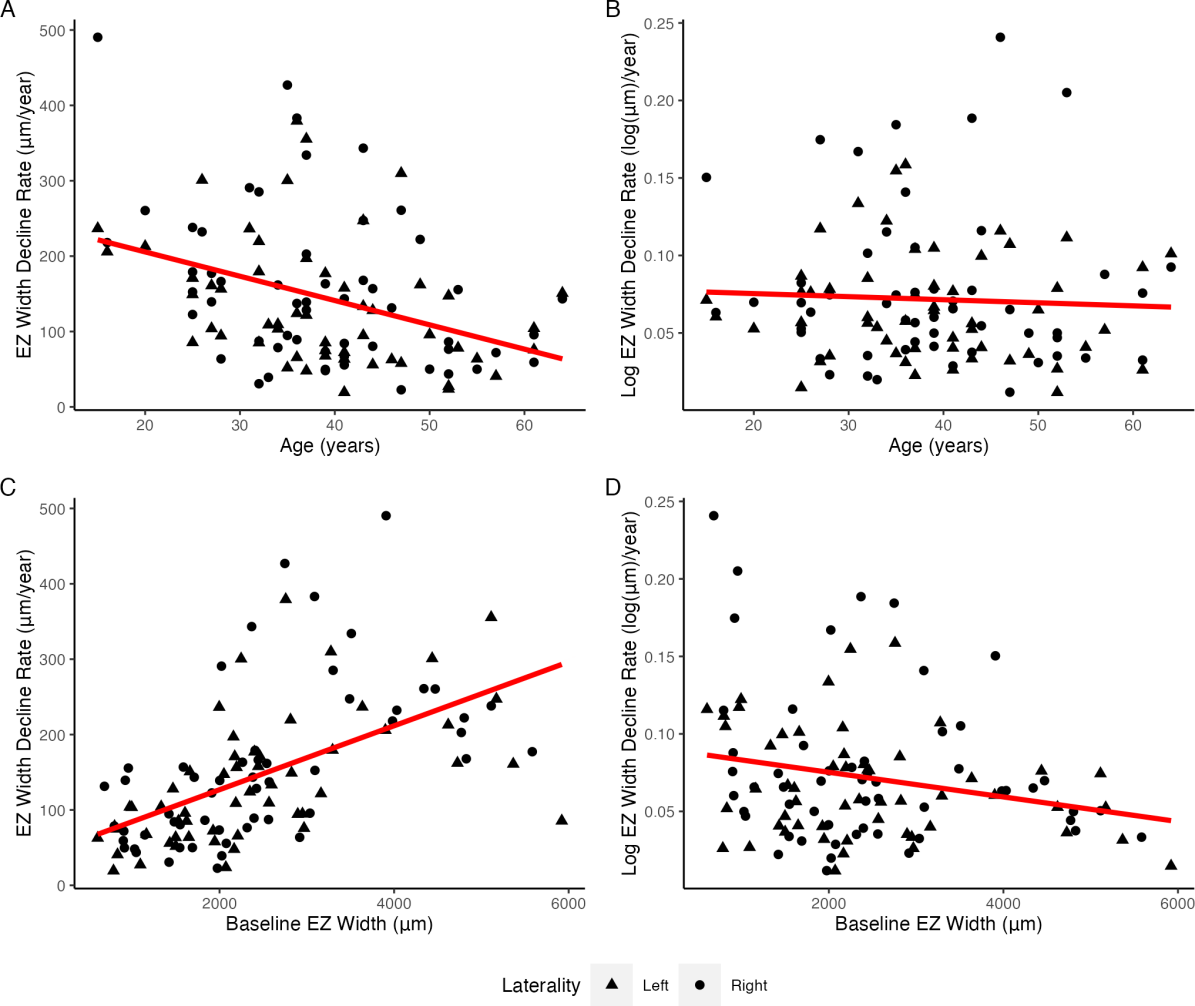
Scatterplots showing the relationship of the ellipsoid zone (EZ) width decline rate with baseline age and EZ width. Solid red lines indicate linear trendlines. (A) EZ width decline rate was correlated with baseline age (Spearman’s correlation coefficient, ρ=−0.41, p<0.001). (B) The use of log-transformed EZ width decline rate reduced the ρ value between decline rate and baseline age to −0.10 (p=0.31). (C) EZ width decline rate was correlated with baseline EZ width (ρ=0.67, p<0.001). (**D**) Using log EZ width reduced the association between decline rate and baseline EZ width (ρ=−0.18, p=0.06).

The EZ width decline rate was negatively correlated with age (Spearman’s correlation coefficient, ρ=−0.41, p<0.001; figure 3A) and positively correlated with baseline EZ width (ρ=0.67, p<0.001; figure 3C). The log-transformation of EZ width reduced the associations of decline rate with both baseline age (ρ=−0.10, p=0.31; figure 3B) and baseline EZ width (ρ=−0.18, p=0.06; figure 3D). Similar associations were observed when left and right eyes were analysed separately (online supplemental table 2B).

The power analysis showed that a 1-year clinical trial using the log-transformed EZ width decline rate as the endpoint would reduce the sample size requirement from 73 participants to 38 participants (48% reduction) compared with using the untransformed EZ width decline rate as the endpoint (online supplemental table 3). Similar reductions were observed when left and right eyes were analysed separately (left: from 63 to 40 eyes with log-transform; right: from 78 to 36 eyes with log-transform).

### Usher syndrome patients exhibit earlier onset of macular EZ width loss

**Figure 4:**
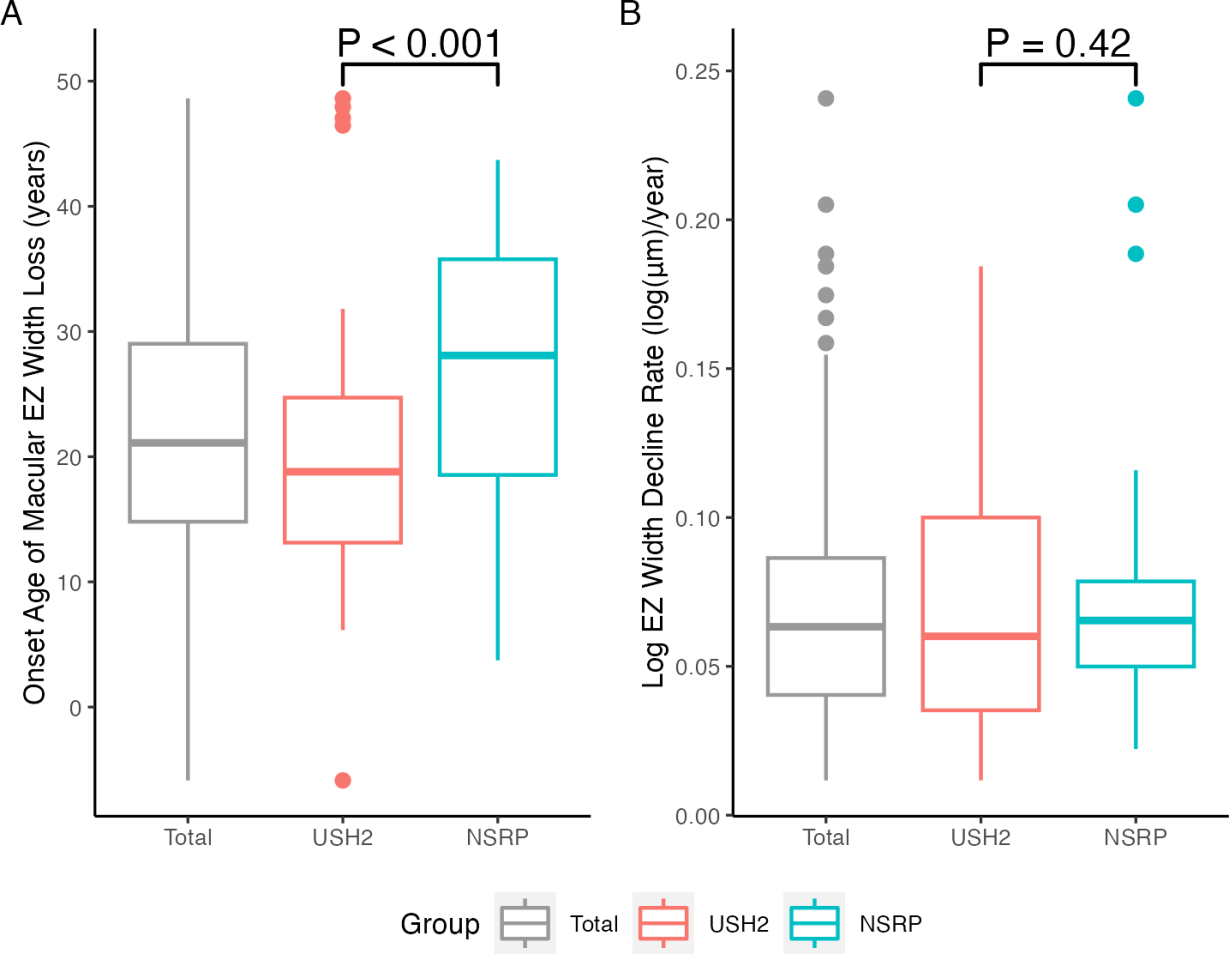
Boxplots depicting estimated onset age of macular ellipsoid zone (EZ) width loss and log EZ width decline rates, stratified by presence of syndromic disease. USH2, n=68 eyes; NSRP, n=42 eyes. (A) Among all 110 eyes, the median and IQR of onset age of macular EZ width loss was 21.1 (14.8–29.0) years. Syndromic eyes exhibited an earlier onset age of macular EZ width loss (18.8 (IQR: 13.1–24.7) years) compared with non-syndromic eyes (28.1 (IQR: 18.5–35.8) years) (p<0.001). (B) Among all eyes, the median log EZ width decline rate was 0.063 (IQR: 0.040–0.086) log (μm)/year. The log EZ width decline rate was comparable between syndromic eyes (0.060 (IQR: 0.035–0.100) log (μm)/year) and non-syndromic eyes (0.065 (IQR: 0.050–0.079) log (μm)/year) (p=0.42). NSRP, non-syndromic retinitis pigmentosa; USH2, Usher syndrome type II.

At baseline, syndromic eyes were younger (36±12 vs 42±10 years, p=0.04; table 1) and exhibited worse visual acuity (0.30 (IQR: 0.18–0.48) vs 0.18 (IQR: 0–0.30) logMAR, p=0.02, table 1) compared with non-syndromic eyes. Syndromic eyes had a 9.3-year earlier median onset age of macular EZ width loss compared with non-syndromic eyes (18.8 (IQR: 13.1–24.7) vs 28.1 (IQR: 18.5–35.8) years, p<0.001; figure 4A) but showed no difference in the median log EZ width decline rate (0.060 (IQR: 0.035–0.100) vs 0.065 (IQR: 0.050–0.079) log (μm)/year, p=0.42; figure 4B). This was equivalent to a median annual EZ width decline rate of 5.83 (IQR: 3.46%–9.52%) and 6.33 (IQR: 4.87%–7.55%), respectively. The results remained unchanged when we analysed the left and right eye separately (online supplemental table 2C).

### Interocular correlations

EZ width measurements across all longitudinal visits were highly correlated between the right and left eyes (Pearson’s correlation coefficient, r=0.96, p<0.001; online supplemental figure 3A) with no significant difference (mean difference±SD=12 ± 348 µm, p=0.65). The predicted onset age of macular EZ width loss was strongly correlated between contralateral eyes (r=0.88, p<0.001; online supplemental figure 3B) with no significant difference in onset age between eyes (mean difference=1.04±5.41 years, p=0.16). The log EZ width decline rate was also well correlated between the right and left eyes although to a lesser degree (r=0.64, p<0.001; online supplemental figure 3C), with no statistically significant difference in rates between eyes (mean difference=0.010±0.039 log (μm)/year, p=0.07).

## Discussion

In *USH2A*-retinopathy, the EZ width and its rate of decline can vary considerably between patients.^8 9 16 27^ Though this could be due to distinct subpopulations of eyes having their own unique patterns of disease progression, a simpler and more unified explanation is that all eyes follow the same overall pattern of progression (linear, quadratic or exponential) but at potentially different rates. If so, the apparent differences in baseline EZ width measurements can be explained by a combination of different entry times (ie, different eyes entered into this natural history study at different times in their disease course) and different rates of disease progression. To adjust for these different entry times and progression rates, we applied BETR to 110 eyes with *USH2A*-retinopathy and synthesised 30 years of inferred disease duration, during which the loss of EZ width was best modelled by exponential decay. Through traditional statistical methods, we showed that the log-transformed EZ width decline rate is a more sensitive endpoint than raw EZ width decline rate; the log transformation increased statistical power (reduced required sample size by 48%) and eliminated the significant dependence on baseline EZ width and patient age. Compared with non-syndromic eyes, syndromic eyes exhibited earlier onset of macular EZ width loss, but comparable rates of log EZ width decline. Finally, we demonstrated the symmetric progression of disease between contralateral eyes.

Our results indicating exponential decline of EZ width are consistent with prior studies showing exponential decline in EZ width, hyperautofluorescent ring size, visual field area and electroretinogram cone response amplitude in various types of RP.^7 8 11 12 28 29^ This exponential decline may reflect a ‘one-hit model’ of cell death where each photoreceptor has a fixed, independent chance of death.^30^ Our observed median annual exponential EZ width decline rate of 6.1% per year was comparable to the 4.5% per year reported in another *USH2A*-specific cohort.^8^ Furthermore, compared with studies of other types of RP, we observed an intermediate rate of EZ width loss, consistent with the current hierarchy of RP disease severity^9 14^ where autosomal recessive disease is believed to progress quicker than autosomal dominant disease (3.4%, <5.4%)^12 13^ but slower than x-linked disease (7%–13.2%).^10 11 13^ Similar to previous studies measuring EZ width or area,^10 31 32^ we found a high degree of symmetry between contralateral eyes.

Notably, we showed that the log EZ width decline rate might be a better endpoint for future clinical trials compared with the raw EZ width decline rate. First, applying the log transformation linearised the exponential decay exhibited by EZ width, which allows for many commonly used statistical tests, including linear regression and linear mixed models. Second, using the log EZ width decline rate required 48% fewer participants to achieve the same statistical power as a similar trial using the raw EZ width decline rate. Finally, whereas the raw EZ width decline rate was significantly correlated with age and baseline EZ width (also seen in prior studies^10 16^), the log EZ width decline rate eliminated these associations with such confounding factors. Therefore, using the log EZ width decline rate reduces the need to stratify or exclude patients based on baseline EZ width or age. These advantages can help relax the complexity and sample size requirements for future clinical trials, which is particularly beneficial for rare diseases such as *USH2A*-retinopathy.

In our comparison of syndromic and non-syndromic disease, we found that the log EZ width declined at similar rates across both groups. To our knowledge, few prior studies have characterised the longitudinal progression of EZ width loss in an *USH2A*-specific cohort stratified by syndromic status. However, our observation of similar progression rates is consistent with analogous studies which found no difference in visual field loss rate^28 33^ or cone spacing rate of change^34^ between syndromic and non-syndromic *USH2A* patients. Therefore, future clinical trials could consider enrolling both syndromic and non-syndromic participants, which further lessens the burden of recruiting adequate numbers of participants. Although syndromic eyes may exhibit lower baseline EZ width^27^ and consequently slower decline of the raw EZ width, these concerns would be negated by using the log EZ width decline rate as the endpoint.

Interestingly, syndromic eyes exhibited an earlier onset of macular EZ width loss. Earlier onset of disease in syndromic *USH2A*-retinopathy has been substantiated through various other endpoints,^27 32 33 35^ but this discrepancy with the rate of macular EZ width loss may underscore essential differences in the mechanism of disease. Assuming that the peripheral loss of EZ width is also similar between syndromic and non-syndromic patients, then the absolute start of EZ loss may occur at an earlier age in syndromic patients. The existence of this ‘critical age,’ as postulated by Massof *et al*, may reflect a triggering event such as UV light, trauma or infection, which initiates the cascade of subsequent photoreceptor death.^36^ The discordance of similar log EZ width decline rates yet dissimilar onset ages of macular EZ width loss suggests that the mechanism for disease *onset* may be different from that of disease progression. Further research is required to uncover the underlying biology of this observation.

We note several limitations of our study. First, we studied a UK-based tertiary centre cohort, which may differ in the type and distribution of *USH2A* variants compared with cohorts from other geographical regions.^35^ Second, the OCT scans used in our analysis were limited to the central 20°. With the advent of widefield OCT, future studies may be able to incorporate patients with earlier stages of the disease. Third, because the relative measurement error of EZ width was higher in eyes with lower baseline EZ width,^16^ this may result in less accurate estimates of decline rates in eyes with advanced diseases. Finally, we did not measure EZ area and were thus unable to assess its performance as an endpoint compared with EZ width.

In conclusion, EZ width displayed exponential decline in *USH2A*-retinopathy across 30 years of synthesised natural history. For future clinical trials, the log EZ width decline rate should be preferred over the raw EZ width decline rate due to greater sensitivity and reduced dependence on baseline characteristics. Lastly, *USH2A*-related syndromic patients exhibited an earlier onset of macular EZ width loss but had comparable rates of log EZ width decline compared with non-syndromic patients.

## supplementary material

10.1136/bjo-2024-325323 online supplemental file 1

## Data Availability

Data are available on reasonable request.
