# Supplementary material for: Long-term natural history of ellipsoid zone width in USH2A-retinopathy
Source: Br J Ophthalmol. 2024 Aug 5;109(3):e325323. doi: 10.1136/bjo-2024-325323 (PMC11866300; doi:10.1136/bjo-2024-325323)
Supplement: online supplemental file 1 [file bjo-109-3-s001.pdf]

Supplementary Figure 1

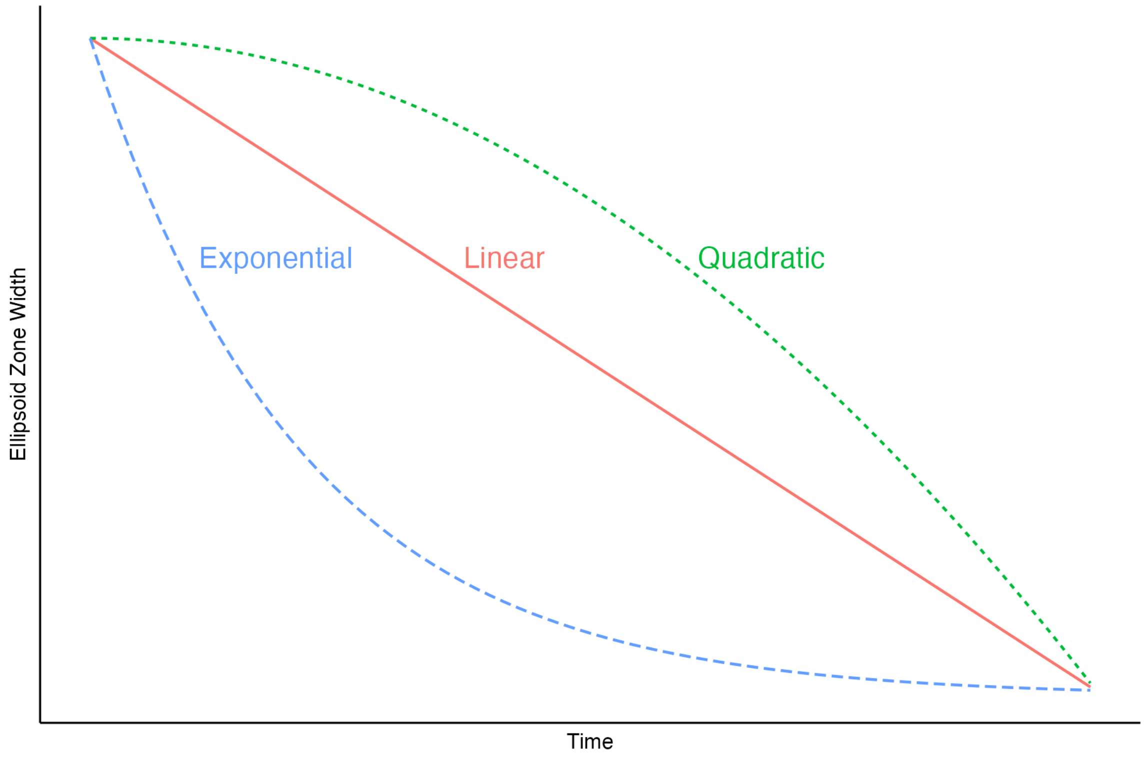

Mathematical models of ellipsoid zone (EZ) width decline analyzed via Bayesian Entry Time Realignment: linear, quadratic, and exponential decay.

Supplementary Figure 2

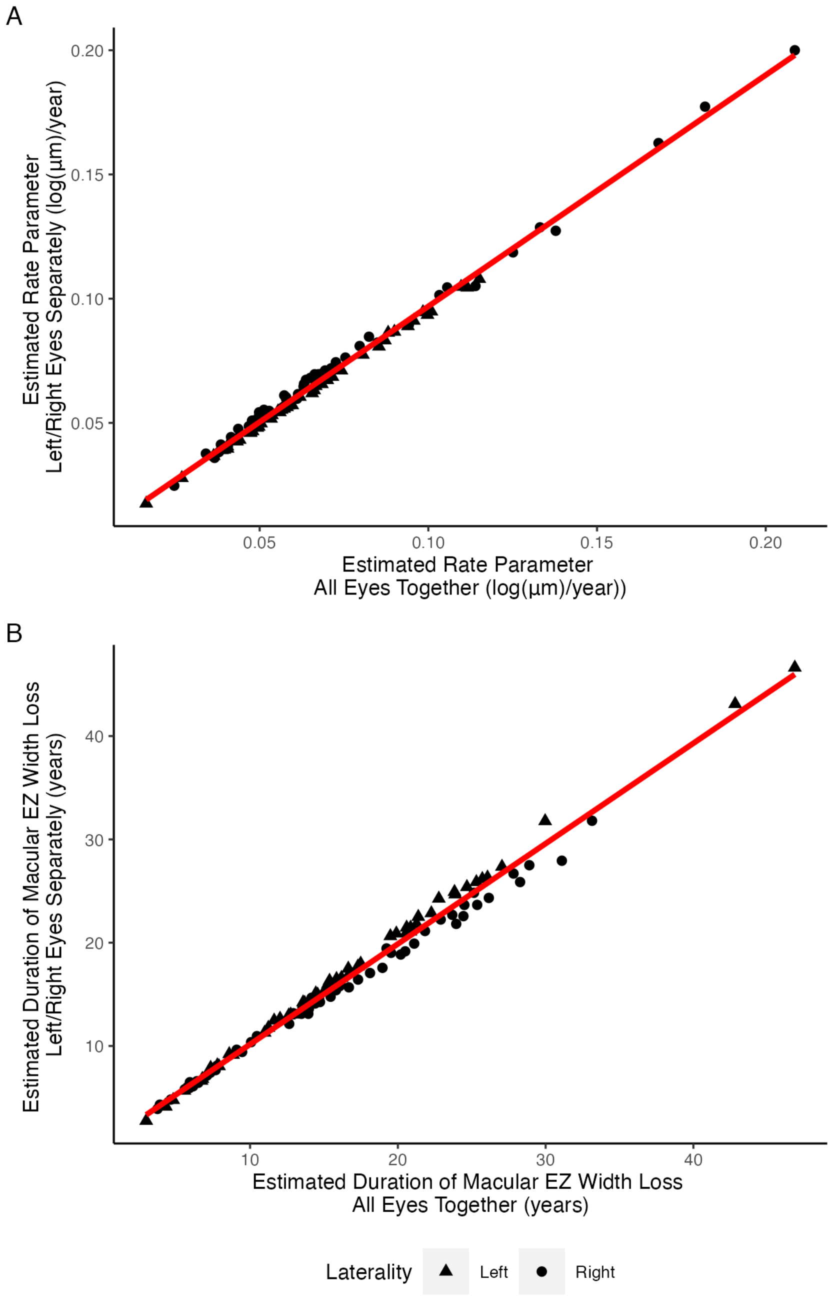

Scatterplots showing sensitivity analysis for BETR-generated parameters, comparing between values generated by analyzing left and right eyes separately (y-axis) and those generated with all eyes combined (x-axis). Trendlines shown in red. (**A**) The BETR-estimated rate parameters (represented by β_1 in the Supplementary Methods) generated by the analysis with all eyes were nearly identical to those generated by analyzing left and right eyes separately (R^2 = 0.99). (**B**) The BETR-estimated durations of macular EZ width loss (represented by δ in the Supplementary Methods) generated by the analysis with all eyes were nearly identical to those generated by analyzing left and right eyes separately (R^2 = 0.99).

Supplementary Figure 3

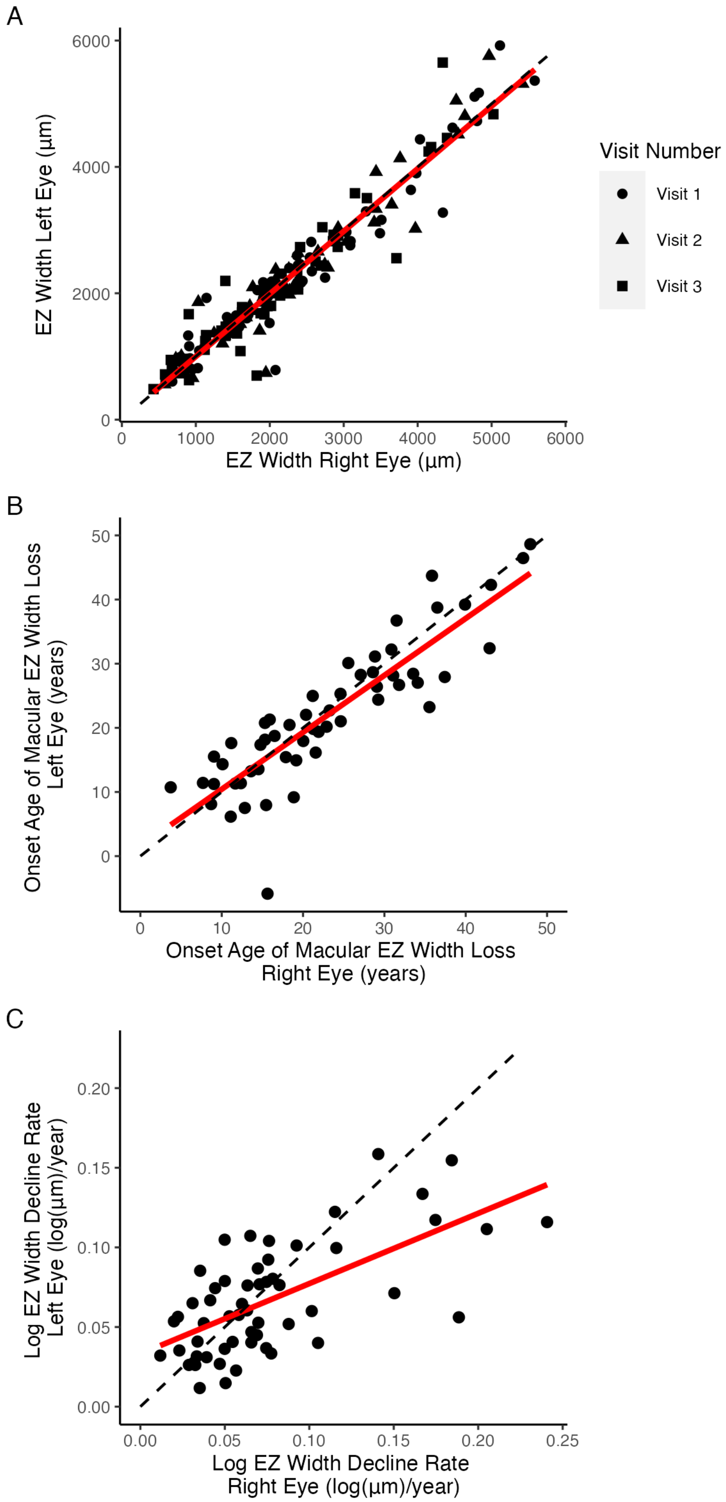

Scatterplots showing the interocular correlations of ellipsoid zone (EZ) width measurements and progression parameters. Dashed black lines indicate lines of equality, solid red lines indicate linear trend lines. (**A**) EZ width measurements were highly correlated between the right and left eyes amongst all visits (Pearson's

correlation coefficient, r = 0.96, $P < 0.001$; difference = 12 ± 348 μm, $P = 0.65$). (**B**) The estimated onset age of macular EZ width loss was highly correlated between contralateral eyes (r = 0.88, $P < 0.001$; difference = 1.04 ± 5.41 years, $P = 0.16$). (**C**) Log EZ width decline rate was well correlated between contralateral eyes but to a lesser extent (r = 0.64, $p < 0.001$; difference = 0.010 ± 0.039 log(μm)/year, $P = 0.07$).

Supplementary Table 1. Allele Variants and Syndromic Status of Participants

Participant	Allele #1	Allele #2	Syndromic (Y/N)
1	c.5836C>T	c.2299delG	Y
2	c.4821G>A	c.1859G>T	Y
3	c.2610C>A	c.2610C>A	Y
4	c.13130C>A	c.1256G>T	Y
5	c.1876C>T	c.2299delG	Y
6	c.14911C>T	c.2299delG	Y
7	c.6967C>T	c.7883dupC	Y
8	c.11864G>A	c.13335_13347delinsCTTG	N
9	c.11700C>A	c.11700C>A	N
10	c.9976C>T	c.9976C>T	N
11	c.100C>T	c.926C>T	N
12	c.7789A>T	c.7789A>T	N
13	c.3841A>T	c.3840G>C	Y
14	c.4474G>T	c.2276G>T	N
15	c.2023C>T	c.2299delG	Y
16	c.8079G>A	c.12575G>A	N
17	c.11065C>T	c.7645_7661del	Y
18	c.8981G>A	c.13274C>T	N
19	c.11156G>T	c.14285A>G	N
20	c.10073G>A	c.920_923dupGCCA	N
21	c.5603T>G	c.10996T>G	N
22	c.10073G>A	c.11156G>A	N
23	c.10561T>C	c.7595-2144A>G	Y
24	c.2802T>G	c.2802T>G	N
25	c.1036A>C	c.13316C>T	Y
26	c.5555A>G	c.13331C>T	N
27	c.13274C>T	c.11875_11876delCA	Y
28	c.6854A>G	c.6653T>C	N
29	c.2276G>T	c.4714C>T	N
30	c.1256G>T	c.13750dupA	Y
31	c.13316C>T	c.2276G>T	N
32	c.2276G>T	c.6470delG	N
33	c.2276G>T	c.14426C>T	N
34	c.4714C>T	c.7595-2144A>G	Y
35	c.10073G>A	c.11549-1G>A	N
36	c.2299delG	c.2299delG	Y

37	c.2299delG	c.2299delG	Y
38	c.2299delG	c.4714C>T	Y
39	c.2299delG	c.7595-2144A>G	Y
40	c.2299delG	c.4714C>T	Y
41	c.1679delC	c.6795_6797delATA	Y
42	c.2299delG	c.1606T>A	Y
43	c.2299delG	c.4510dup	Y
44	c.13374delA	c.2276G>T	N
45	c.2299delG	c.5614_5620delGCTGTCG	Y
46	c.2299delG	c.1256G>T	Y
47	c.11694delC	c.3158-6A>G	Y
48	c.12819A>T	c.1055T>A	Y
49	c.9860_9873delATGATG GCCATGGC	c.6730G>A	Y
50	c.2299delG	c.4714C>T	Y
51	c.2081G>A	c.4714C>T	Y
52	c.4714C>T	c.14426C>T	Y
53	c.2299delG	c.2299delG	Y
54	c.2299delG	c.4714C>T	Y
55	c.895delC	c.2994A>T	Y

Supplementary Table 2A. Sensitivity Analysis of DIC Scores from BETR Models Stratified by Eye Laterality

	Right Eye	Left Eye	Both Eyes
Linear decline model, DIC	-391	-451	-839
Quadratic decline model, DIC	-319	-307	-614
Exponential decline model, DIC	-649	-703	-1379

BETR = Bayesian Entry Time Realignment, DIC = Deviance Information Criterion

Supplementary Table 2B. Sensitivity Analysis of Spearman Correlation Analysis for EZ Width Decline Rates and Baseline Features Stratified by Eye Laterality

	**Right Eye**		**Left Eye**		**Both Eyes**	
	ρ	**P**	ρ	**P**	ρ	**P**
EZ width decline rate vs Baseline age	-0.37	0.005	-0.44	< 0.001	-0.41	< 0.001
Log EZ width decline rate vs Baseline age	-0.09	0.53	-0.10	0.46	-0.10	0.31
EZ width decline rate vs Baseline EZ width	0.68	< 0.001	0.66	< 0.001	0.67	< 0.001
Log EZ width decline rate vs Baseline EZ width	-0.17	0.22	-0.22	0.1	-0.18	0.06

EZ = ellipsoid zone, ρ = Spearman's correlation coefficient

Supplementary Table 2C. Sensitivity Analysis of EZ Width Progression Parameters Stratified by Eye Laterality

	Left Eye			Right Eye			Both Eyes		
	USH2	NSRP	P	USH2	NSRP	P	USH2	NSRP	P
Log EZ width decline rate, log(μm)/year, median [IQR]	0.055 [0.033 - 0.096]	0.067 [0.052 - 0.079]	0.32	0.064 [0.041 - 0.100]	0.065 [0.044 - 0.077]	0.89	0.060 [0.035 - 0.100]	0.065 [0.050 - 0.079]	0.42
Onset age of macular EZ width loss, years, median [IQR]	18.3 [13.3 - 24.8]	27.9 [19.8 - 32.4]	0.01	19.4 [13.0 - 24.6]	30.9 [18.3 - 35.9]	0.01	18.8 [13.1 - 24.7]	28.1 [18.5 - 35.8]	< 0.001

EZ = ellipsoid zone, IQR = interquartile range, NSRP = nonsyndromic retinitis pigmentosa, USH2 = Usher syndrome type II

Supplementary Table 3. Power Characteristics of EZ Width Decline Rate Endpoints

	EZ width decline rate (μm/year)		Log EZ width decline rate (log(μm)/year)	
**Number of eyes from dataset available for analysis**	**Mean (SD)**	**Number of eyes needed[a]**	**Mean (SD)**	**Number of eyes needed**
70	207 (154)	73	0.092 (0.052)	38

SD = standard deviation

[a]The number of eyes needed per group for a trial powered to detect a 30% decrease in the EZ width decline rate (or the corresponding log EZ width decline rate) at one year, assuming a significance of 0.05, power of 80%, and a 1:1 enrollment ratio between treatment and control groups.

Supplementary Methods

Formally, the three progression models can be represented mathematically by the following equations (1-3), where EZ_0 represents initial EZ width at disease onset, t represents time in years since baseline clinical visit, β_1 represents the magnitude of disease progression (rate parameter), and δ represents the predicted interval in years between disease onset and baseline clinical visit (duration parameter).

$$(1)\ Linear\ Decline: EZ = EZ_0 - \beta_1(t+\delta)$$

$$(2)\ Quadratic\ Decline: EZ = EZ_0 - \beta_1(t+\delta)^2$$

$$(3)\ Exponential\ Decline: EZ = EZ_0 e^{-\beta_1(t+\delta)}$$

Here, we defined disease onset as the onset of macular EZ width loss, corresponding to the time point in which the EZ width = 6000 μm (approximately the maximum detectable width in a twenty-degree OCT scan). We set EZ_0 to this value for all three models. To implement BETR, we supplied the EZ width and time point of each visit and allowed the algorithm to calculate values of β_1 and δ for each eye. We then subtracted δ from the age of each eye at its baseline visit to calculate the onset age of macular EZ width loss.

Because BETR can only evaluate models whose outputs are strictly positive, we first applied the following mathematical transformations to the linear (4) and quadratic (5) models before implementing BETR. We subtracted EZ width from 6000 μm to obtain EZ^*, defined as the width of the EZ lost since onset of OCT-detectable disease.

$$(4)\ Linear\ Decline: EZ^* = EZ_0 - EZ = \beta_1(t+\delta)$$

$$(5)\ Quadratic\ Decline: EZ^* = EZ_0 - EZ = \beta_1(t+\delta)^2$$

BETR is applied using Markov chain Monte Carlo methods. We used the following settings in our analysis: each model was run with a burn-in period of 100,000 iterations, followed by posterior sampling of 1.5 million iterations (500,000 iterations each from 3 chains) which was thinned by a factor of 10. Posterior inference was conducted via posterior medians, and we assessed for convergence of each model using Geweke's diagnostic and by inspection of the trace plots generated from the posterior samples.
